# The application of enhanced recovery after surgery for upper gastrointestinal surgery: Meta-analysis

**DOI:** 10.1186/s12893-019-0669-3

**Published:** 2020-01-03

**Authors:** Zhen-Dong Huang, Hui-Yun Gu, Jie Zhu, Jie Luo, Xian-Feng Shen, Qi-Feng Deng, Chao Zhang, Yan-Bing Li

**Affiliations:** 1 Center for Evidence-Based Medicine and Clinical Research, Taihe Hospital, Hubei University of Medicine, No.32, South Renmin Road, Shiyan, 442000 China; 2 Department of Orthopedic, Zhongnan Hospital of Wuhan University, Wuhan University, Wuhan, 430000 China; 3 Trade Union, Taihe Hospital, Hubei University of Medicine, Shiyan, 442000 China; 4 Department of General Surgery, Taihe Hospital, Hubei University of Medicine, No.32, South Renmin Road, Shiyan, 442000 China

**Keywords:** Enhanced recovery after surgery, Multimodal perioperative care, Upper gastrointestinal surgery, Gastric cancer, Postoperative morbidity

## Abstract

**Background:**

Although enhanced recovery after surgery (ERAS) has made great progress in the field of surgery, the guidelines point to the lack of high-quality evidence in upper gastrointestinal surgery.

**Methods:**

Randomized controlled trials in four electronic databases that involved ERAS protocols for upper gastrointestinal surgery were searched through December 12, 2018. The primary endpoints were lung infection, urinary tract infection, surgical site infection, postoperative anastomotic leakage and ileus. The secondary endpoints were postoperative length of stay, the time from end of surgery to first flatus and defecation, and readmission rates. Subgroup analysis was performed based on the type of surgery.

**Results:**

A total of 17 studies were included. The results of the meta-analysis indicate that there was a decrease in rates of lung infection (RR = 0.50, 95%CI: 0.33 to 0.75), postoperative length of stay (MD = -2.53, 95%CI: − 3.42 to − 1.65), time until first postoperative flatus (MD = -0.64, 95%CI: − 0.84 to − 0.45) and time until first postoperative defecation (MD = -1.10, 95%CI: − 1.74 to − 0.47) in patients who received ERAS, compared to conventional care. However, other outcomes were not significant difference. There was no significant difference between ERAS and conventional care in rates of urinary tract infection (*P* = 0.10), surgical site infection (*P* = 0.42), postoperative anastomotic leakage (*P* = 0.45), readmissions (*P* = 0.31) and ileus (*P* = 0.25).

**Conclusions:**

ERAS protocols can reduce the risk of postoperative lung infection and accelerating patient recovery time. Nevertheless, we should also consider further research ERAS should be performed undergoing gastrectomy and esophagectomy.

## Background

The concept of enhanced recovery after surgery (ERAS) [1] was first introduced by the pioneer surgeon H. Kehlet in 1997. ERAS has been of considerable interest to the field of medicine in recent years [2]. ERAS protocols were first applied in colorectal surgery [2], expanding gradually to obstetrics and gynaecology [3], urology [4], and pelvic surgery [5]. Multimodal perioperative care played a vital role in the ERAS protocols that were based on ERAS society guidelines [6]. Lower complication rates, faster gastrointestinal function recovery, faster free activity, lower average hospitalization costs and shorter postoperative hospital stays were observed in patients in the ERAS group. ERAS has adopted a series of measures to reduce the physical and psychological trauma that surgical patients experience, and these help the patients rapidly achieve functional recovery.

The upper part of the gastrointestinal tract includes the esophagus, stomach, and duodenum. A scientific paper about cases of cancer around the world [7] showed that digestive cancer has the highest morbidity and mortality rate of all cancers. Surgery is the mainstay of treatment for digestive cancer [8]. The purpose of surgery is to completely eliminate the primary tumour and to rebuild the digestive tract. Given that it is traumatic, surgery itself is the most common source of stress for surgical patients.

Even though a meta-analysis of observational studies of postoperative complication outcome [9] was done in 2017, it did not give a detailed list that classified the complications. A meta-analysis [10] in 2018 indicated that the ERAS protocol increased the rate of readmissions in elderly patients with gastric cancer, but this result requires more studies to confirmits findings, it is only for the analysis of Gastrectomy. In this meta-analysis, we conducted a comprehensive evaluation of the effect of ERAS on upper gastrointestinal surgery patients across RCTs. The aim of this study was to evaluate the impact of ERAS protocols for upper gastrointestinal surgery postoperative complications and postoperative recovery time for clinical ERAS practice and provide more evidence for the update of the guideline.

## Methods

### Literature search

We used the guidelines from the Preferred Reporting Items for Systematic Review and Meta-Analyses [11]. All studies were obtained by searching Ovid Medline, Ovid EMBASE, CENTRAL and ISIWeb of Science for articles that were published through December 12, 2018. Detailed search strategies are shown in Additional file 1.

### Inclusion and exclusion criteria

Studies were included when they met the following criteria: (1) human patients undergoing gastric surgery, esophagectomy or duodenectomy; (2) intervention used: ERAS protocols; (3) comparison: conventional care; (4) outcomes evaluated: postoperative lung infection (LI), postoperative urinary tract infection (UTI), postoperative surgical site infection (SSI), postoperative anastomotic leakage and ileus, readmission rate, postoperative length of stay (PLOS), time from surgery to first flatus and defecation; types of postoperative complications according to original study authors’ definition (5) study design: RCTs. No minimum sample size or minimum number of ERAS process measures was required for inclusion. Studies were excluded for the following reasons: (1) non-full-text English article; (2) emergency surgery; (3) sleeve gastrectomy for obesity; (4) data was inadequate for meta-analysis; (5) ERAS protocols that were not followed for the entire perioperative period.

### Data extraction and quality assessment

According to the inclusion and exclusion criteria, two authors (ZDH and QFD) independently selected the studies to be included by reading abstracts and full-text articles. If a disagreement arose due to inconsistent understanding, then consensus was reached by arbitration and discussion with a third investigator. Information and data were extracted by two independent authors (XFS and YF) and checked for accuracy by a third investigator (CZ). The following information was extracted from all of the trials: first author, year of publication, patients’ characteristics (i.e., age and sex), surgical type, ERAS protocol interventions, and follow-up time. Primary endpoints for the study included major incidents of the following complications: LI, UTI, SSI, postoperative anastomotic leakage and ileus. Secondary endpoints were PLOS, the time until intestinal function recovery (i.e., time until the first flatus and defecation), and readmission rates.

Two authors (ZDH and QFD) independently assessed the risk of biasin accordance with the Cochrane risk of bias tool [12]. The risk of bias in each item was graded as “high risk”, “low risk” or “unclear”.

### Statistical analysis

Statistical meta-analysis was performed with R software (meta software package). The Doi plots [13] were drawn by MetaXL (Version 5.3). Pooled risk ratios (RR) with 95% confidence intervals (CI) wereapplied to analyse dichotomous data; continuous data were analysed as the mean difference (MD) with a 95%CI. However, many studies only reported the median and range of the samples or the first and third quartiles. In these cases, we needed to estimate the sample mean and SD [14, 15]. We used the converted sample mean and SD for meta-analysis. I^2^ [16] statistics were used to assess the heterogeneity of each analysis. If I^2^ > 40 [16], we assumed that there was statistical heterogeneity. Meanwhile, the pooled effect size was calculated by the random effects model (REM). For studies with zero events in their arms, this was done by adding a fixed value (typically 0.5) to all cells [17]. Subgroup analysis was performed based on the type of cancer, surgical procedure and scope of gastrectomy.

Doi plots were used to evaluate the data for possible publication bias. Doi plots are a new method of graphing that are used to detectpossible publication bias and have a higher sensitivity than funnel plots. An LFK index within ±1 indicates that the Doi plots haveno asymmetry; when the LFK index exceeds ±1 but is within ±2, it indicates that the Doi plots have minor asymmetry; when the LFK index exceeds ±2, it suggests that there is major asymmetry.

## Results

### Literature identification

**Fig. 1:**
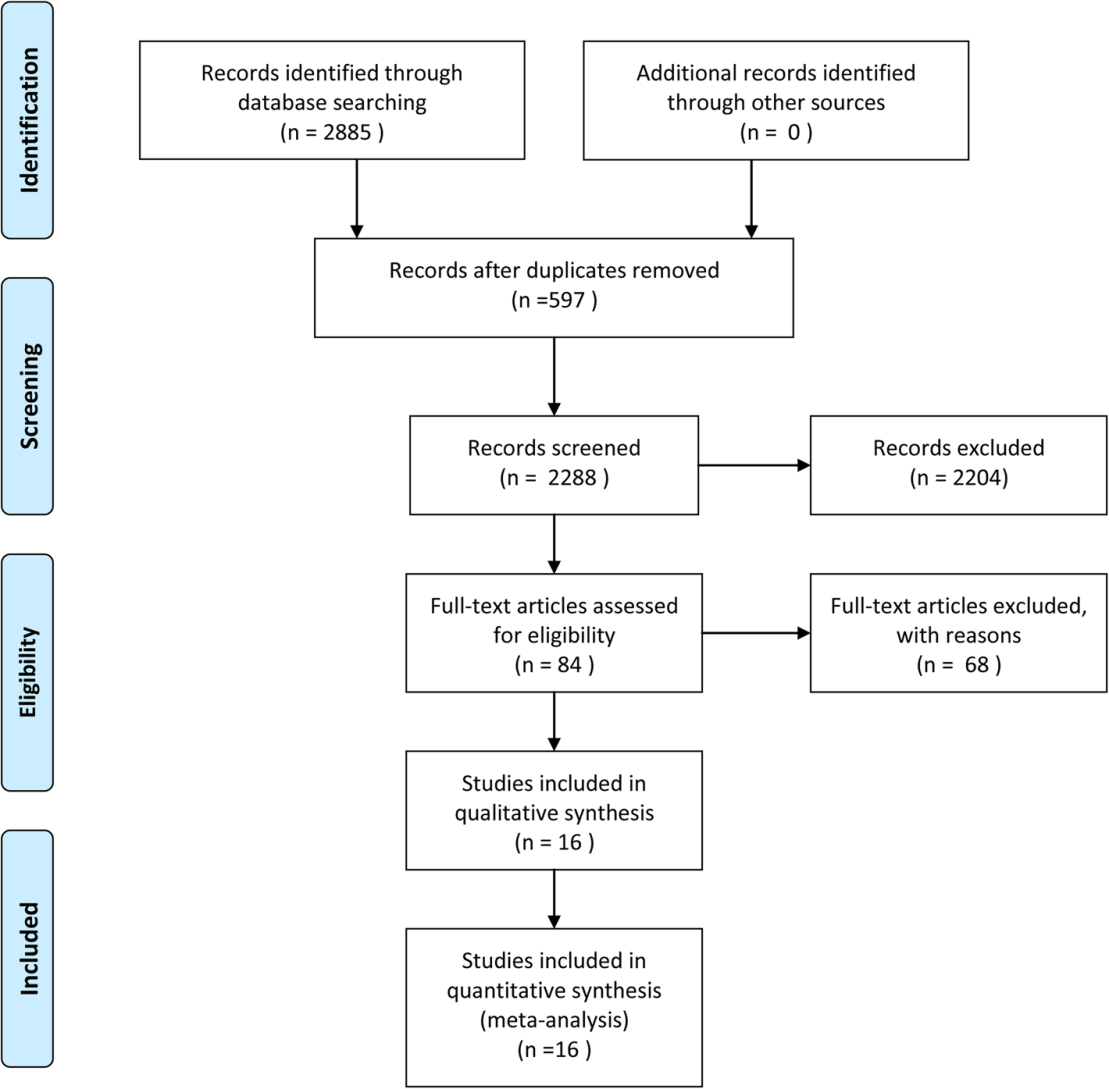
PRISMA flow diagram

Ovid Medline, Ovid EMBASE, CENTRAL and Web of Science were systematically searched through December 12, 2018. The search resulted in 2885 articles. After initial evaluation, 597 studies were removed for being duplicates, 2204 for being irrelevant (as determined by reading the abstracts), and 67 studies were excluded for reasons determined by reading the full text (Additional file 2). 16 studies [18–33] were included in the final meta-analysis. Figure 1 shows the work flow for the selection of studies.

### Study and ERAS characteristics

Studies were included in the meta-analysis when they adhered to consensus guidelines for ERAS protocols [6, 34]. The basic characteristics of the included studies are shown in Table 1. Table 2 shows the details of the key elements of ERAS protocols for all of the studies, including the type of disease and the surgical site. It also summarizes ERAS protocol items, and it details the primary endpoints and follow-up times. Two studies reported comparing laparoscopic to open surgery [19, 23]; the other reported on patients aged 45 to 74 years and 75 to 89 years [21]. Finally, a total of 19 RCTs from 16 studies, included 1830 patients, of whom 907 were in the ERAS arm and 923 were in the control arm, were found to be studies that compared ERAS to conventional care. Gastric cancer surgery was reported in 14 RCTs from 11 studies, and esophagectomy was reported in 5 studies.

**Table 1:** Main characteristics of the included studies

Study	Year	Sample (n)		Age (years)		Sex, male/female		Follow-up (weeks)
		ERAS	CC	ERAS	CC	ERAS	CC	
Wang [18]	2010	45	47	58.8 ± 9.7	56.9 ± 9.1	32/13	29/18	4
Chen [LS] [19]	2012	19	22	59(49–71)	62.5(45–72)	10/9	10/12	4
Chen [OS] [19]	2012	21	20	62.5(45–72)	64.5(49–75)	9/12	12/8	4
Feng [20]	2013	59	60	55 ± 11.4	55.8 ± 10.1	41/18	44/16	4
Bu [45-74y] [21]	2015	64	64	62.4 ± 7.8	63 ± 7.4	31/33	35/29	4
Bu [75-89y] [21]	2015	64	64	80.1 ± 4	79.6 ± 3.5	37/27	40/24	4
Abdikarim [22]	2015	30	31	63 ± 12	62 ± 11	21/9	20/11	4
Liu [LS] [23]	2016	21	21	69.2 ± 5.1	70.3 ± 5.8	10/11	12/9	4
Liu [OS] [23]	2016	21	21	67.8 ± 3.9	68.6 ± 4.9	9/12	11/10	4
Fujikuni [24]	2016	40	40	< 70(29), ≥70(11)	< 70(28), ≥70(12)	20/20	24/16	4
Tanaka [25]	2017	73	69	68(29–85)	67(44–85)	49/24	49/20	4
Xia [26]	2017	73	76	61 (40–75)	63 (35–75)	48/25	50/26	4
Wu [27]	2017	34	41	63.74 ± 9.56	62.93 ± 9.44	25/9	31/10	NR
Kim [28]	2012	22	22	52.6 ± 11.69	57.5 ± 14.5	13/9	15/7	2
Zhao [29]	2014	34	34	55.14 ± 10.65	57.86 ± 11.34	27/7	25/9	4
Chen [30]	2016	128	132	56.34 ± 13.28	55.72 ± 10.34	103/25	106/26	4
Li [31]	2017	55	55	67.73 ± 6.69	67 ± 5.58	38/17	41/14	8
Zhang [32]	2017	47	47	45–76	45–75	28/19	25/22	12
Zhang [33]	2018	57	57	66.89 ± 13.45	67.01 ± 12.78	39/18	38/19	NR

Note: *ERAS* Enhacned recovery after surgery, *CC* Conventional care, *LS* Laparoscopic surgery, *OS* Open surgery, *NR* Not reported; 45-74y: Patients aged 45–74 years; 75-89y: Patients aged 75–89 years

**Table 2:** The Items of Characteristics of Included Studies from Enhanced Recovery After Surgery (ERAS) and Controls

Author, Year	Surgery	Disease	I	N	Enhanced Recovery After Surgery Interventions																		Number of item using ERAS
					①	②	③	④	⑤	⑥	⑦	⑧	⑨	⑩	⑪	⑫	⑬	⑭	⑮	⑯	⑰	⑱	
Wang 2010 [18]	Gastrectomy	Gastric Cancer	E	45	✓	✓	✓	✓			✓	✓		✓		✓	✓	✓	✓	✓	✓	✓	14
			C	47																			
Chen (LS) 2012 [19]	Distal Gastrectomy	Gastric Cancer	E	19	✓	✓	✓	✓			✓	✓				✓	✓	✓		✓	✓	✓	12
			C	22				✓			✓												
Chen (OS) 2012 [19]	Distal Gastrectomy	Gastric Cancer	E	21	✓	✓	✓	✓				✓				✓	✓	✓			✓	✓	10
			C	20				✓															
Feng 2013 [20]	Radical gastrectomy	Gastric Cancer	E	59			✓	✓		✓		✓			✓		✓				✓	✓	8
			C	60				✓															
Bu (45-74y) 2015 [21]	Gastrectomy	Gastric Cancer	E	64	✓	✓	✓			✓	✓	✓			✓	✓	✓	✓			✓	✓	12
			C	64	✓					✓													
Bu (75-89y) 2015 [21]	Gastrectomy	Gastric Cancer	E	64	✓	✓	✓			✓	✓	✓			✓	✓	✓	✓			✓	✓	12
			C	64	✓					✓													
Abdikarim 2015 [22]	Radical gastrectomy	Stomach Carcinomas	E	30	✓	✓	✓	✓			✓	✓				✓					✓	✓	9
			C	31	✓						✓												
Liu (LS) 2016 [23]	Radical gastrectomy	Gastric Cancer	E	21	✓	✓				✓	✓	✓				✓		✓			✓	✓	9
			C	21							✓												
Liu (OS) 2016 [23]	Radical gastrectomy	Gastric Cancer	E	21	✓	✓				✓		✓				✓		✓			✓	✓	8
			C	21																			
Fujikuni 2016 [24]	Gastrectomy	Gastric Cancer, Submucosal Tumor	E	40			✓				✓					✓					✓		4
			C	40							✓												
Tanaka 2017 [25]	Gastrectomy	Gastric Cancer	E	73		✓	✓			✓	✓								✓		✓	✓	7
			C	69							✓											✓	
Xia 2017 [26]	Radical Gastrectomy	Gastric Cancer	E	73	✓	✓	✓				✓	✓			✓	✓	✓	✓	✓		✓	✓	12
			C	76							✓												
Wu 2017 [27]	Distal Gastrectomy	Gastric Cancer	E	34	✓	✓	✓					✓			✓	✓	✓	✓			✓	✓	10
			C	41																			
Kim 2012 [28]	Distal Gastrectomy	Gastric Cancer	E	22	✓	✓	✓				✓	✓			✓			✓	✓		✓	✓	10
			C	22		✓					✓	✓						✓					
Zhao 2014 [29]	Esophagectomy	Esophageal Cancer	E	34	✓		✓	✓				✓		✓	✓		✓	✓			✓	✓✓	10
			C	34	✓																		
Chen 2016 [30]	Esophagectomy	Esophageal Cancer	E	128	✓				✓			✓		✓	✓		✓	✓	✓		✓	✓	10
			C	132	✓																		
Li 2017 [31]	Esophagectomy	Esophageal Cancer	E	55	✓						✓				✓			✓				✓	5
			C	55							✓												
Zhang 2017 [32]	Esophagectomy	Esophageal Cancer combined with Metabolic Syndrome	E	47	✓						✓									✓	✓	✓	5
			C	47							✓												
Zhang 2018 [33]	Esophagectomy	Esophageal Carcinoma	E	57	✓	✓	✓		✓						✓			✓			✓		7
			C	57	✓				✓														
			C	37	✓		✓							✓	✓								

Note: *E/ERAS* Enhacned recovery after surgery, *C* Conventional care, *LS* Laparoscopic surgery, *OS* Open surgery; 45-74y: Patients aged 45–74 years; 75-89y: Patients aged 75–89 years; I: Intervention; PLOS: Postoperative length of hospital stays; N: Number of patients; Enhanced Recovery After Surgery items: ①Preoperative counselling; ②Avoid Bowel preparation; ③Preoperative Carbohydrate; ④No Preanesthetic Medication; ⑤Antithrombotic prophylaxis; ⑥Antimicrobial Prophylaxis; ⑦Minimal Invasive Surgery; ⑧Avoid Nasogastric intubation; ⑨Avoid Postoperative nausea and vomiting; ⑩Epidural analgesia; ⑪Avoiding hypothermia; ⑫Fluid balance; ⑬Avoid Peritoneal Drainage; ⑭Early Urinary drainage Removal; ⑮ Regional Analgesia; ⑯Stimulation of bowel movement; ⑰Early Oral Feeding; ⑱ Early mobilization

### Quality assessment

The results of the quality assessment are shown in Additional file 3. It is notable that none of the 19 RCTs can blind the surgeon or the patient during the surgery. In addition, all 19 RCTs were quite similar in their risk of bias. Nine of the RCTs did not report random sequence generation, only 2 RCTs had blinded outcome assessments.

### Primary outcomes

#### Lung infection

**Fig. 2:**
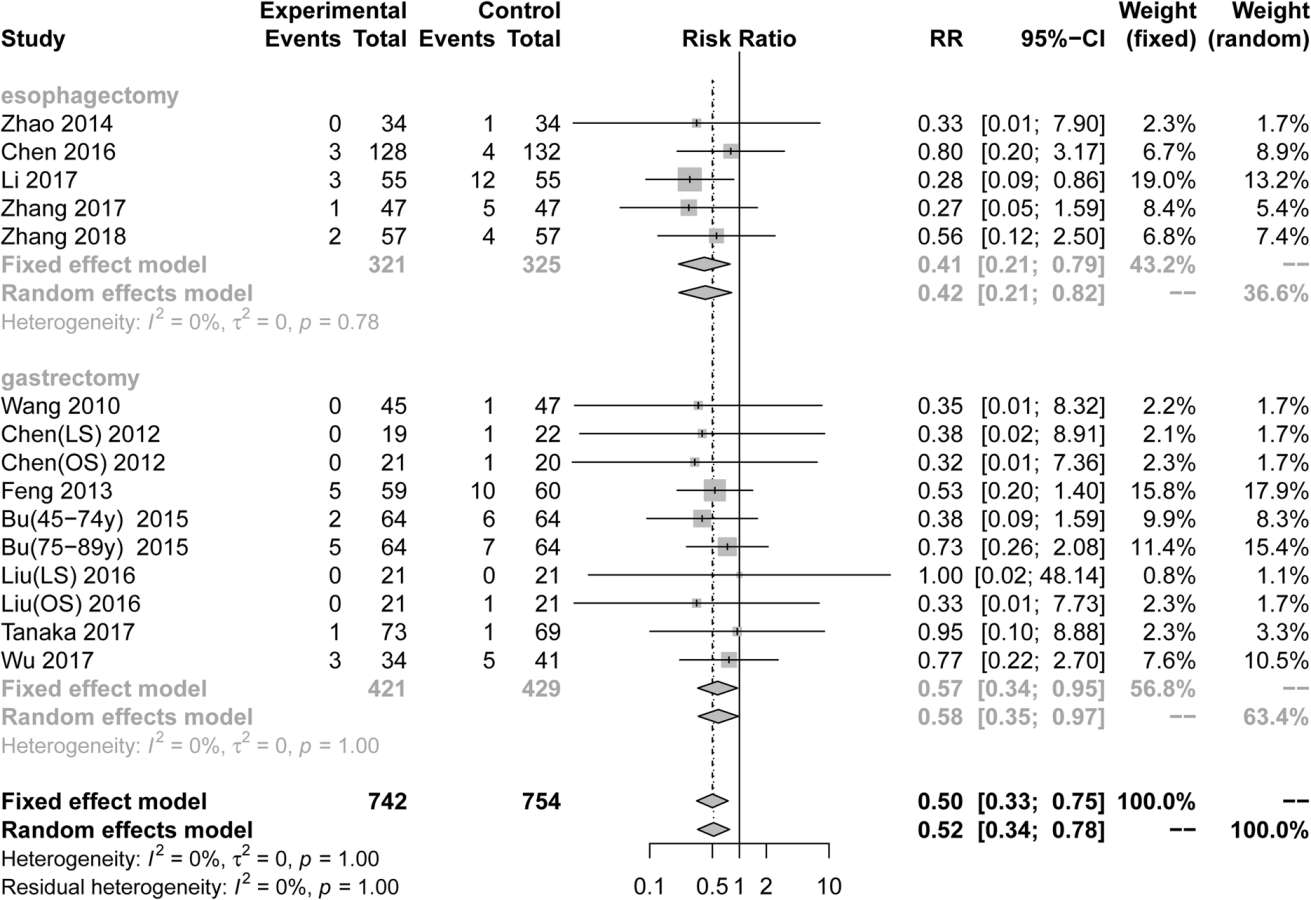
Forest plot evaluating postoperative lung infection between ERAS and conventional care

Fifteen RCTs including 1496 patientsreported postoperative LI. Pooling the resultssuggested that ERAS protocols significantly decreased the incidence of postoperative LI compared to conventional care (Fig. 2, RR = 0.50, 95%CI: 0.33 to 0.75). The test of heterogeneity (I^2^ = 0%) indicated that there was little heterogeneity among these trials.

Among RCTs performed in the area of gastric surgery, analysis indicated that the incidence of LI after surgery was significantly decreased by using ERAS protocols (Fig. 2, RR = 0.57, 95%CI: 0.34 to 0.95, I^2^ = 0%). Among RCTs performed in the area of esophagectomies, the incidence of LI after surgery was significantly reduced by using ERAS protocols (Fig. 2, RR = 0.41, 95%CI: 0.21 to 0.79, I^2^ = 0%). However, based on subgroup analyses of the surgical procedure and scope of gastrectomy, Table 3 showed that there were no statistical differences in all subgroup analyses of LI.

**Table 3:** The results of subgroup analyses based on the surgical procedure and scope of gastrectomy

Outcomes	Scope of gastrectomy			Surgical procedure of gastrectomy		
	Radical gastrectomy	Distal Gastrectomy	Mix	Laparoscopic surgery	Open surgery	Mix
Lung infection	RR = 0.51, 95%CI[0.18; 1.40]	RR = 0.35, 95%CI[0.04; 3.22]	RR = 0.62, 95%CI[0.33; 1.15]	RR = 0.72, 95%CI[0.19; 2.81]	RR = 0.54, 95%CI[0.29; 1.01]	RR = 0.54, 95%CI[0.15; 1.95]
Urinary tract infection	RR = 0.33, 95%[0.01; 8.02]	RR = 0.98, 95%CI[0.14; 6.63]	RR = 0.58, 95%CI[0.29; 1.19]	RR = 2.14, 95%CI[0.20; 23.49]	RR = 0.5, 95%CI[0.21; 1.21]	RR = 0.60, 95%CI[0.20; 1.80]
Surgical site infection	RR = 0.51, 95%CI[0.10; 2.74]	RR = 0.95, 95%CI[0.06; 14.22]	RR = 0.94, 95%CI[0.45; 1.96]	RR = 0.84, 95%CI[0.36; 1.95]	RR = 0.77, 95%CI[0.18; 3.37]	RR = 0.97, 95%CI[0.25; 3.75]
Postoperative anastomotic leakage	RR = 0.34, 95%CI[0.04; 3.25]	RR = 1.05, 95%CI[0.07; 16.17]	RR = 1.21, 95%CI[0.53; 2.74]	RR = 2.56, 95%CI[0.39; 16.93]	RR = 1.15, 95%CI[0.42; 3.10]	RR = 0.97, 95%CI[0.23; 4.18]
Postoperative ileus	RR = 0.34, 95%CI[0.04; 3.25]	RR = 1.05, 95%CI[0.07; 16.17]	RR = 1.95, 95%CI[0.95; 4.02]	RR = 0.69, 95%CI[0.12; 4.01]	RR = 2.13, 95%CI[0.95; 4.81]	RR = 1.00, 95%CI[0.23; 4.29]
Postoperative length of stay	MD = -1.79, 95%CI[−2.59; −0.99]	MD = -1.64, 95%CI[−2.60; −0.33]	MD = -1.88, 95%CI[−2.63;-1.12]	MD = -1.95, 95%CI[−2.99; −0.91]	MD = -1.83, 95%CI[−3.01; −0.66]	MD-1.36, 95%CI[−1.70; −1.03]
Flatus	MD = -0.75, 95%CI[−1.09; − 0.41]	MD = -0.45, 95%CI[− 0.62; − 0.28]	MD = -0.83, 95%CI[−1.22;-0.45]	MD = -0.81, 95%CI[− 2.04; 0.43]	MD = -0.68, 95%CI[− 1.08; − 0.27]	MD = -0.59, 95%CI[− 0.83; − 0.35]
Defecation	MD = -1.63, 95%CI[− 2.79; − 0.47]	Not applicable	MD = -0.54, 95%CI[− 0.86; − 0.22],	MD = -1.36, 95%CI[− 3.05; 0.34]	MD = -1.05, 95%CI[− 1.45; − 0.65]	MD = -0.65, 95%CI[− 1.28; − 0.02]
Readmission rates	RR = 1.02, 95%CI[0.02; 50.41]	RR = 3.00, 95%CI[0.13; 69.79]	RR = 1.99, 95%CI[1.04; 3.82]	RR = 1.13, 95%CI[0.32; 4.08]	RR = 2.64, 95%CI[1.20; 5.81]	RR = 0.95, 95%Ci[0.06; 14.82]

#### Urinary tract infection

**Fig. 3:**
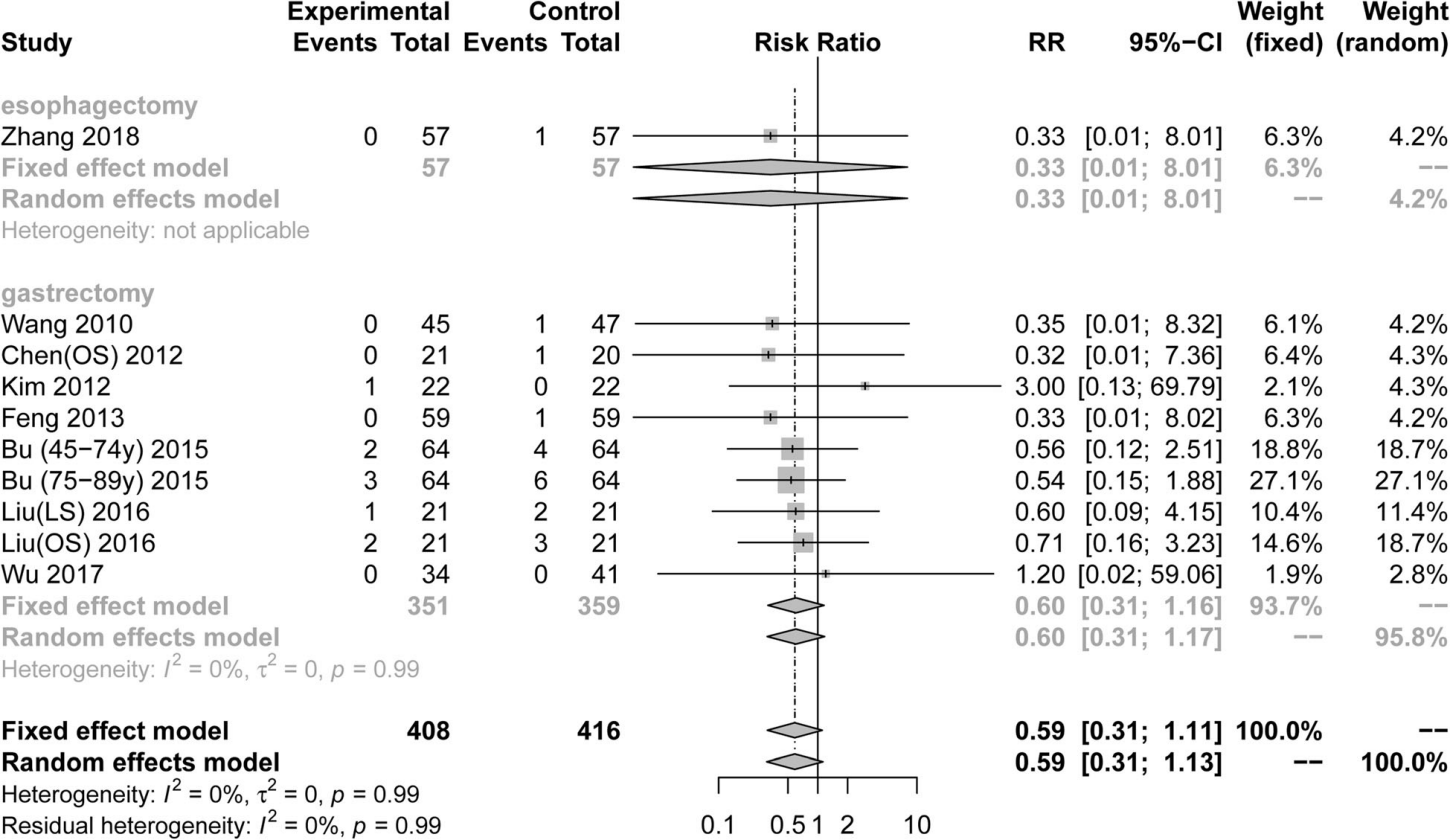
Forest plot evaluating postoperative urinary tract infection between ERAS and conventional care

Ten RCTs included 824 patients diagnosed with postoperative UTI. Pooling the results suggested that ERAS protocols did not increase the incidence of urinary tract infection compared to conventional care (Fig. 3, RR = 0.59, 95% CI: 0.31 to 1.11). The test of heterogeneity (I^2^ = 0%) indicated that there was little heterogeneity among these trials.

Among RCTs performed in the area of gastric surgery, analysis indicated that the incidence of postoperative UTI was not increased by ERAS protocols (Fig. 3, RR = 0.60, 95%CI: 0.31 to 1.16, I^2^ = 0%). There were too few RCTs about esophagectomies to calculatethe incidence of postoperative UTI in this area. However, the results of subgroup analyses of UTI based on the surgical procedure and scope of gastrectomy were no statistical differences in Table 3.

#### Surgical site infection

**Fig. 4:**
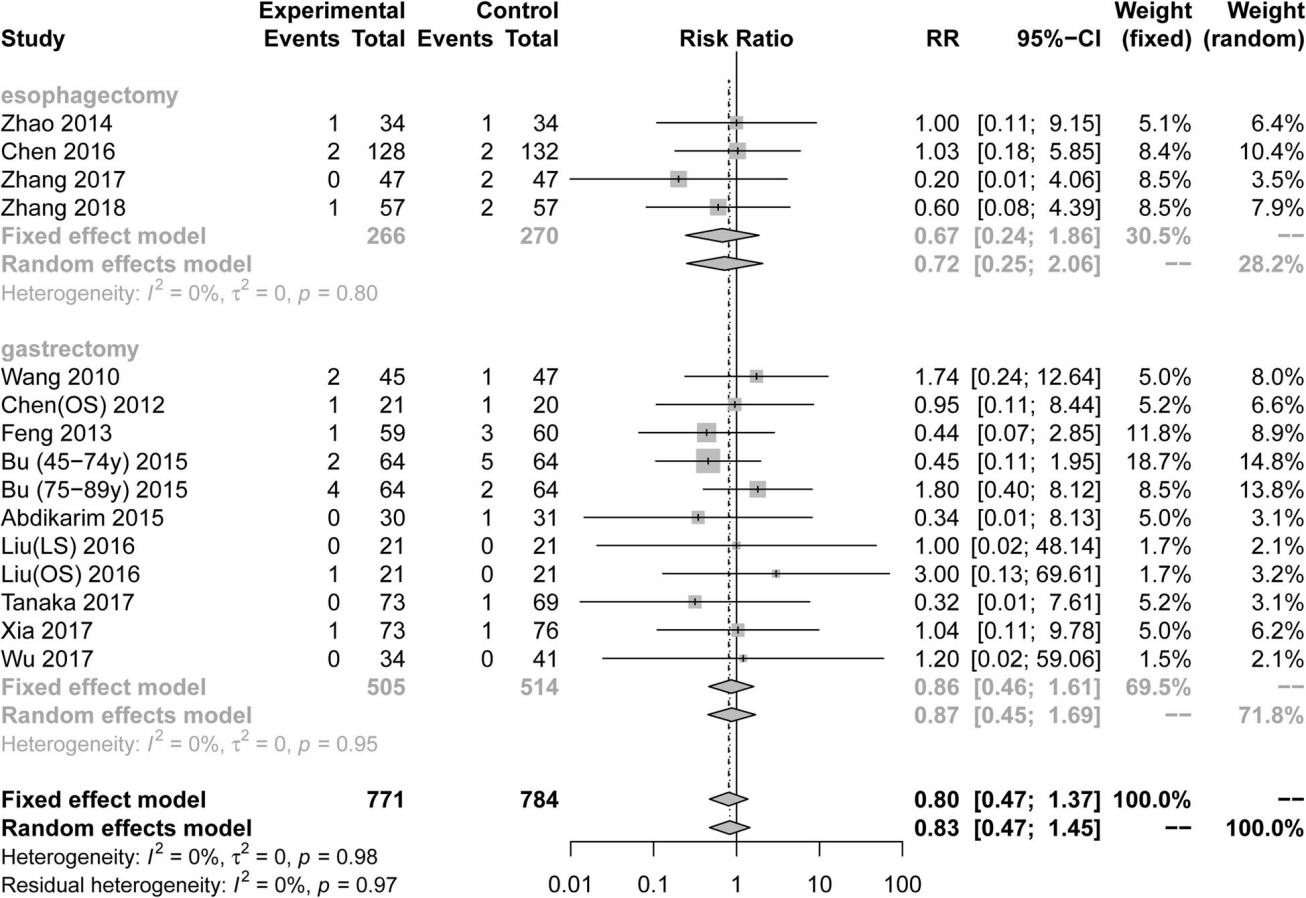
Forest plot evaluating postoperative surgical site infection between ERAS and conventional care

Fifteen RCTs included 1555 patients who reported postoperative SSI. Pooling the resultssuggested that ERAS protocols did not increase the incidence of postoperative SSI compared to conventional care (Fig. 4, RR = 0.80, 95%CI: 0.47 to 1.37). The test of heterogeneity (I^2^ = 0%) indicated that there was little heterogeneity among these trials.

Among RCTs performed in the area of gastric surgery, analysis indicated that the incidence of SSI after surgery was not increased by ERAS protocols (Fig. 4, RR = 0.86, 95%CI: 0.46 to 1.61, I^2^ = 0%). Among RCTs performed in the area of esophagectomiesy surgery, the incidence of SSI was not increased (Fig. 4, RR = 0.67, 95%CI: 0.24 to 1.86, I^2^ = 0%). However, Table 3 demonstrated that there were no statistical differences in all subgroup analyses of SSI based on the surgical procedure and scope of gastrectomy.

#### Postoperative anastomotic leakage

**Fig. 5:**
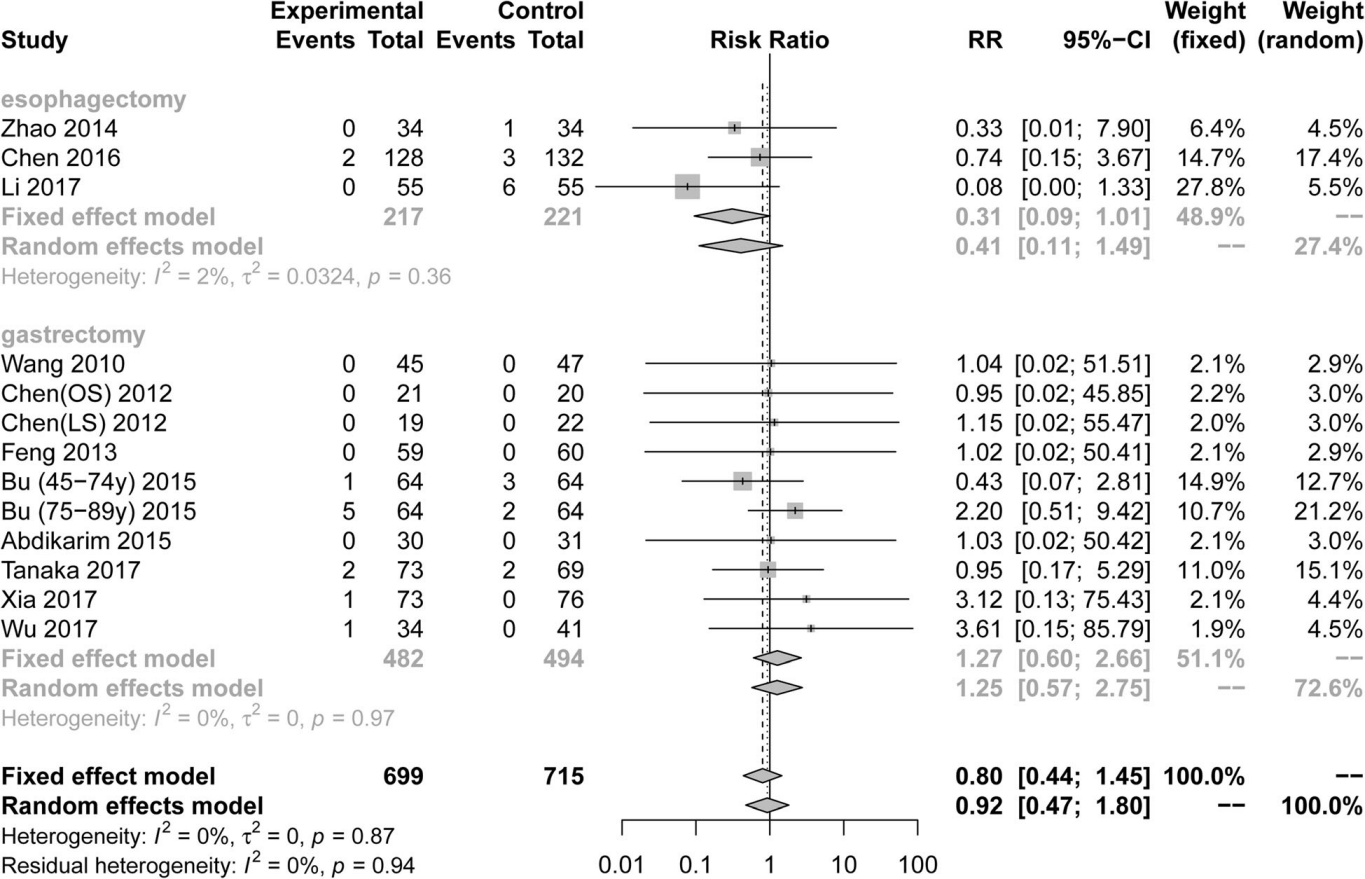
Forest plot evaluating postoperative anastomotic leakage between ERAS and conventional care

Fourteen RCTs including 1414 patients reported postoperative anastomotic leakage. Pooling the results suggested that ERAS protocols did not increase the incidence of postoperative anastomotic leakage compared to conventional care (Fig. 5, RR = 0.80, 95%CI: 0.44 to 1.45). The test of heterogeneity (I^2^ = 0%) indicated that there was little heterogeneity among these trials.

Among RCTs performed in the area of gastric surgery, analysis indicated that the incidence of anastomotic leakage after surgery was not increased by ERAS protocols (Fig. 5, RR = 1.27 95%CI: 0.60 to 2.66, I^2^ = 0%). Among RCTs performed in the area of esophagectomies, postoperative anastomotic leakage (Fig. 5, RR = 0.31, 95%CI: 0.09 to 1.01, I^2^ = 2%) was not increased by ERAS protocols. However, there were no statistical differences in all subgroup analyses of postoperative anastomotic leakage based on the surgical procedure and scope of gastrectomy in Table 3.

#### Postoperative ileus

**Fig. 6:**
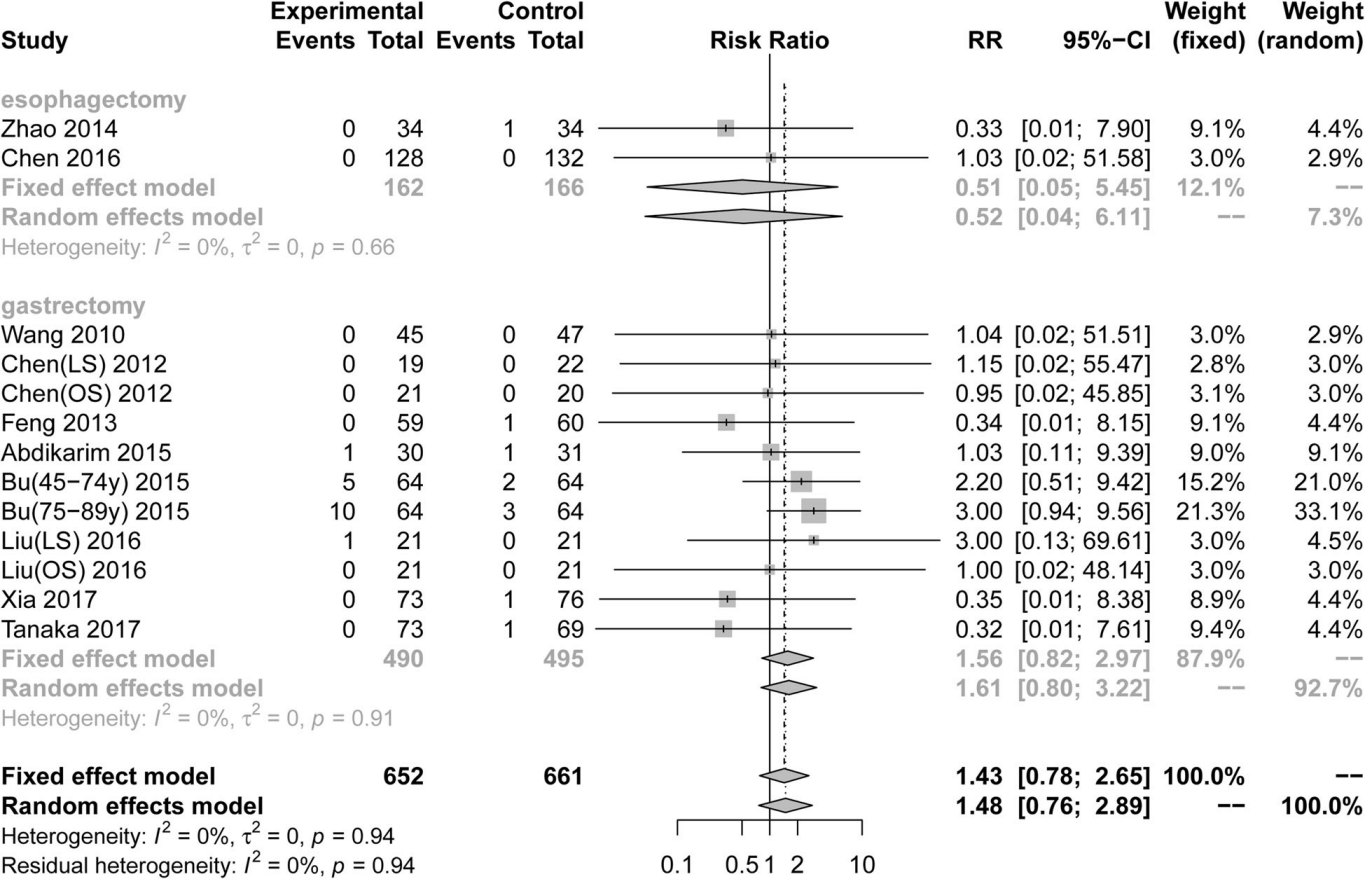
Forest plot evaluating postoperative ileus between ERAS and conventional care

Thirteen RCTs (1313 patients) reported postoperative ileus. Pooling the results suggested that ERAS protocols did not increase the incidence of postoperative ileus compared to conventional care (Fig. 6, RR = 1.43, 95%CI: 0.78 to 2.65). The test of heterogeneity (I^2^ = 0%) indicated that there was little heterogeneity among these trials.

Among RCTs performed in the area of gastric surgery, analysis indicated that the incidence of ileus after surgery was not increased by ERAS protocols (Fig. 6, RR = 1.56, 95%CI: 0.82 to 2.97, I^2^ = 0%). In the area of esophagectomies, postoperative anastomotic leakage (Fig. 6, RR = 1.56, 95%CI: 0.82 to 2.97, I^2^ = 0%) was not increased by ERAS protocols. However, the results that there were no statistical differences were found in all subgroup analyses of postoperative anastomotic leakage based on the surgical procedure and scope of gastrectomy (Table 3).

### Secondary outcomes

**Postoperative length of stay.**

**Fig. 7:**
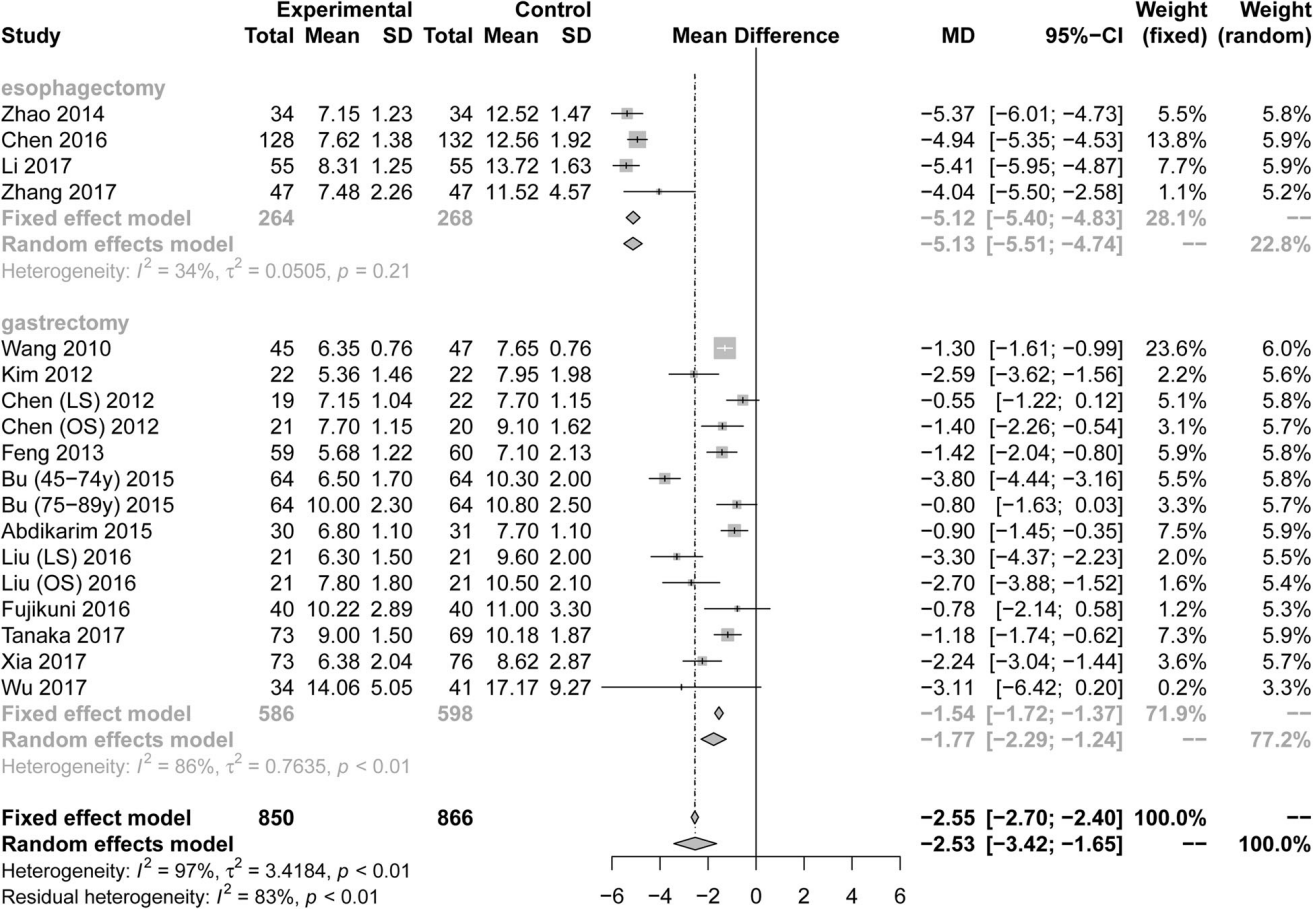
Forest plot evaluating postoperative PLOS between ERAS and conventional care

Eighteen RCTs (1716 patients) reported PLOS. Pooling the results suggested that ERAS protocols significantly decreased the postoperative length of stay compared to conventional care (Fig. 7, MD = − 2.53, 95%CI: − 3.42 to − 1.65). The test of heterogeneity (I^2^ = 97%) indicated that there was a high degree of heterogeneity among these trials.

Among RCTs performed in the area of gastric surgery, analysis indicated that PLOS was significantly reduced by ERAS protocols (Fig. 7, MD = − 1.77, 95%CI = − 2.29 to − 1.24, I^2^ = 85.8%). Among RCTs performed in the area of esophagectomies, PLOS was significantly reduced by ERAS protocols (Fig. 7, MD = − 5.12, 95%CI: − 5.40 to − 4.83, I^2^ = 34%). Based on the surgical procedure and scope of gastrectomy, all subgroup analyses in PLOS, including radical gastrectomy (MD = − 1.79, 95%CI: − 2.59 to − 0.99), distal gastrectomy (MD = − 1.64, 95%CI: − 2.60 to − 0.33), laparoscopic surgery (MD = − 1.95, 95%CI: − 2.99 to − 0.91), and open surgery (MD = − 1.83, 95%CI: − 3.01 to − 0.66), showed statistical differences by ERAS protocols in Table 3.

### The duration of intestinal function recovery

**Fig. 8:**
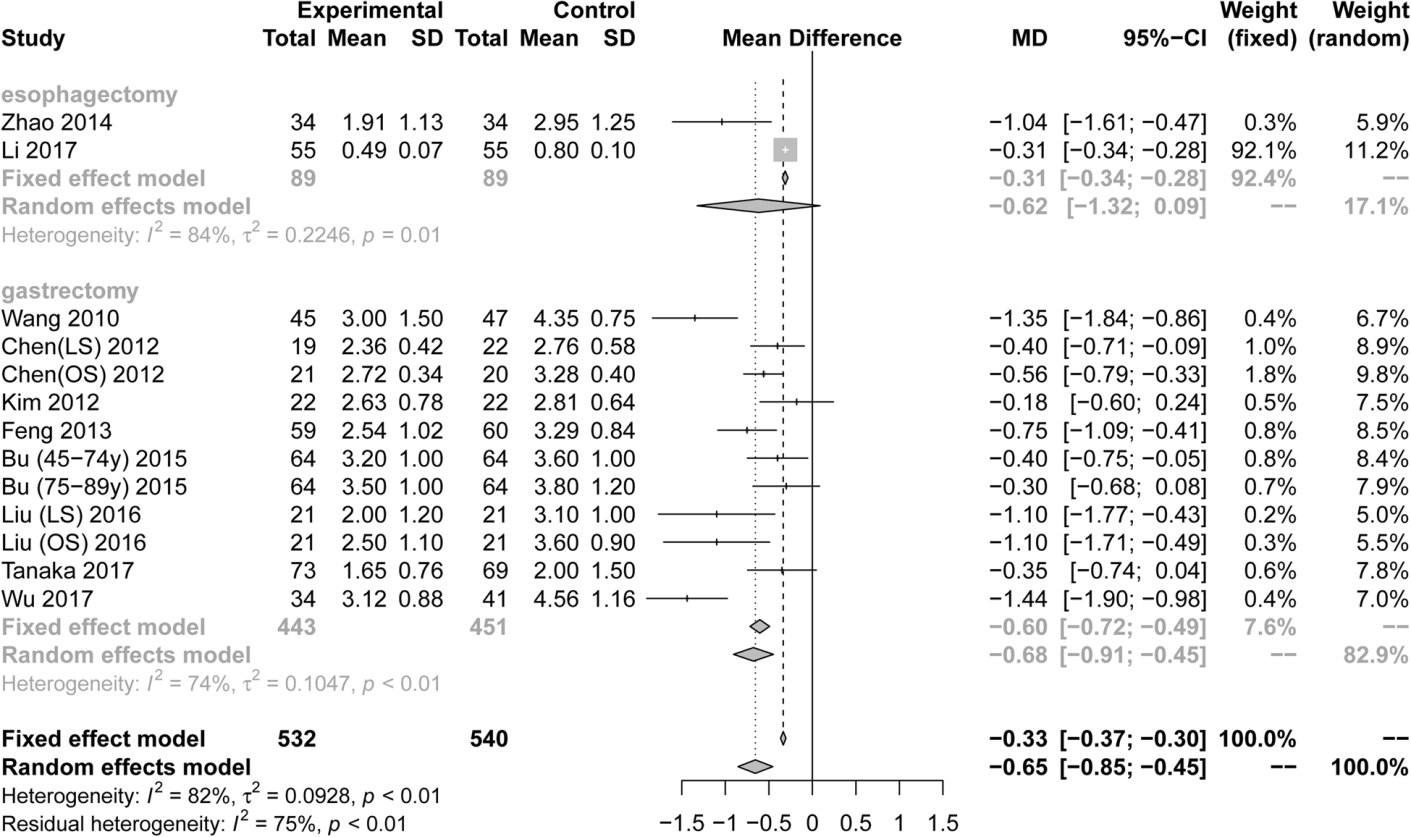
Forest plot evaluating the durtion of the first flatus between ERAS and conventional care

Thirteen RCTs (1072 patients) reported the time until the first postoperative flatus. Pooling the results suggested that ERAS protocols significantly decreased the time until the first postoperative flatus compared to conventional care (Fig. 8, MD = − 0.65, 95% CI: − 0.85 to − 0.45). The test of heterogeneity (I^2^ = 82%) indicated that there was significant heterogeneity among these trials.

**Fig. 9:**
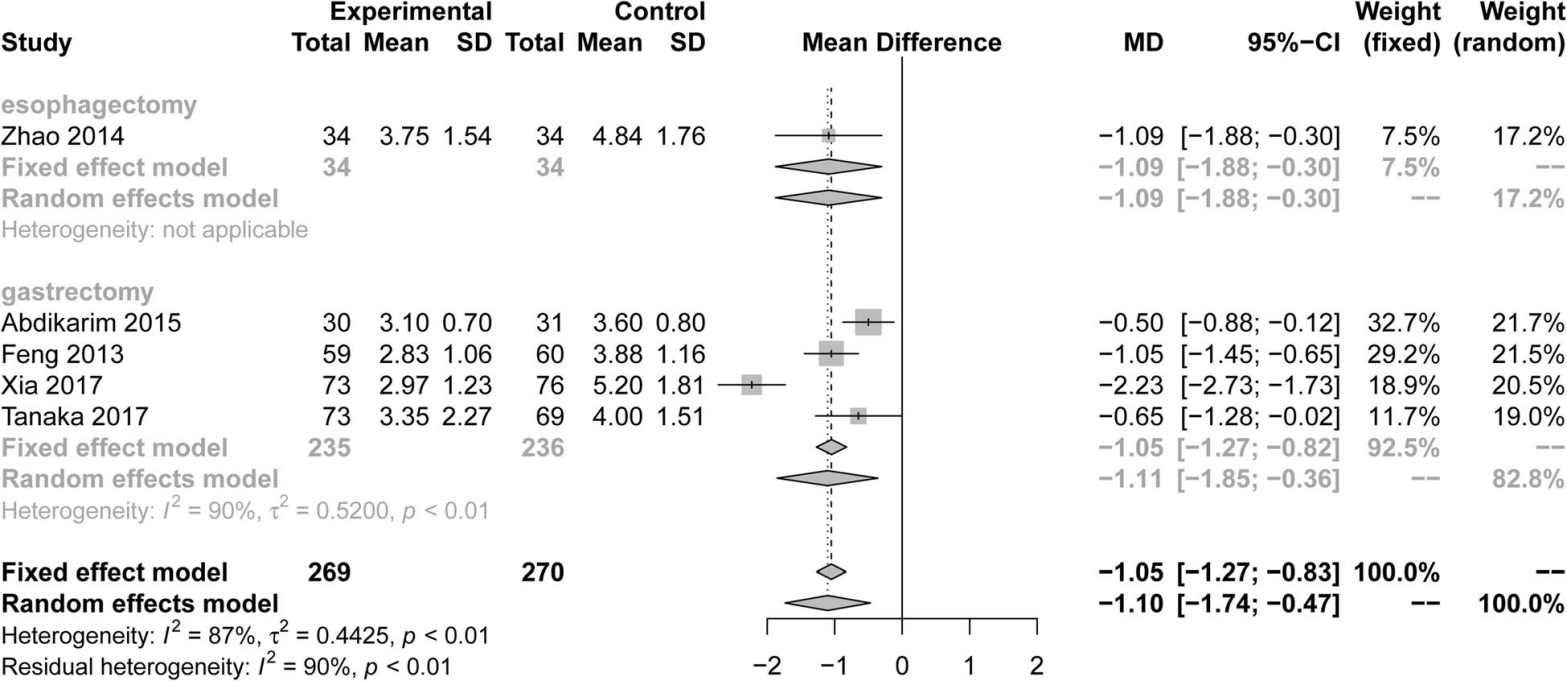
Forest plot evaluating the durtion of the first defecation between ERAS and conventional care

Five RCTs (539 patients) reported the time until the first postoperative defecation. Pooling the results suggested that ERAS protocols significantly decreased the time until the first postoperative defecation compared to conventional care (Fig. 9, MD = − 1.10, 95% CI: − 1.74 to − 0.47). The test of heterogeneity (I^2^ = 87%) indicated that there was significant heterogeneity among these trials.

Among RCTs performed in the area of gastric surgery, analysis indicated that ERAS was associated with a significant reduction in the time until the first postoperative flatus (Fig. 8, MD = − 0.68, 95%CI: − 0.85 to − 0.45, I^2^ = 74%) and until the first postoperative defecation (Fig. 9, MD = − 1.11, 95%CI: − 1.85 to − 0.36, I^2^ = 90%). However, for the esophagectomies, ERAS protocols significantly decreased the time until the first postoperative defecation (Fig. 9, MD = − 1.09, 95%CI: − 1.88 to − 0.30, I^2^ = Not applicable), but it wasn’t decreased the time the first postoperative flatus (Fig. 8, MD = − 0.62, 95%CI: − 1.32 to 0.09, I^2^ = 84%). Based on the surgical procedure and scope of gastrectomy, the subgroup analyses of first postoperative flatus, including radical gastrectomy (MD = − 0.75, 95%CI: − 1.09 to − 0.41), distal gastrectomy (MD = − 0.45, 95%CI: − 0.62 to − 0.28), and open surgery (MD = − 0.599, 95%CI: − 1.08 to − 0.27), showed statistical differences by ERAS protocols, but laparoscopic surgery (MD = − 0.81, 95%CI: − 2.04 to 0.43) wasn’t statistical differences in Table 3. Subgroup analyses of first postoperative defecation, including radical gastrectomy (MD = − 1.63, 95%CI: − 2.79 to − 0.47) and open surgery (MD = − 1.05, 95%CI: − 1.45 to − 0.65), showed statistical differences by ERAS protocols, but laparoscopic surgery (MD = − 1.36, 95%CI: − 3.05 to 0.34) wasn’t statistical differences in Table 3.

### Readmission rates

**Fig. 10:**
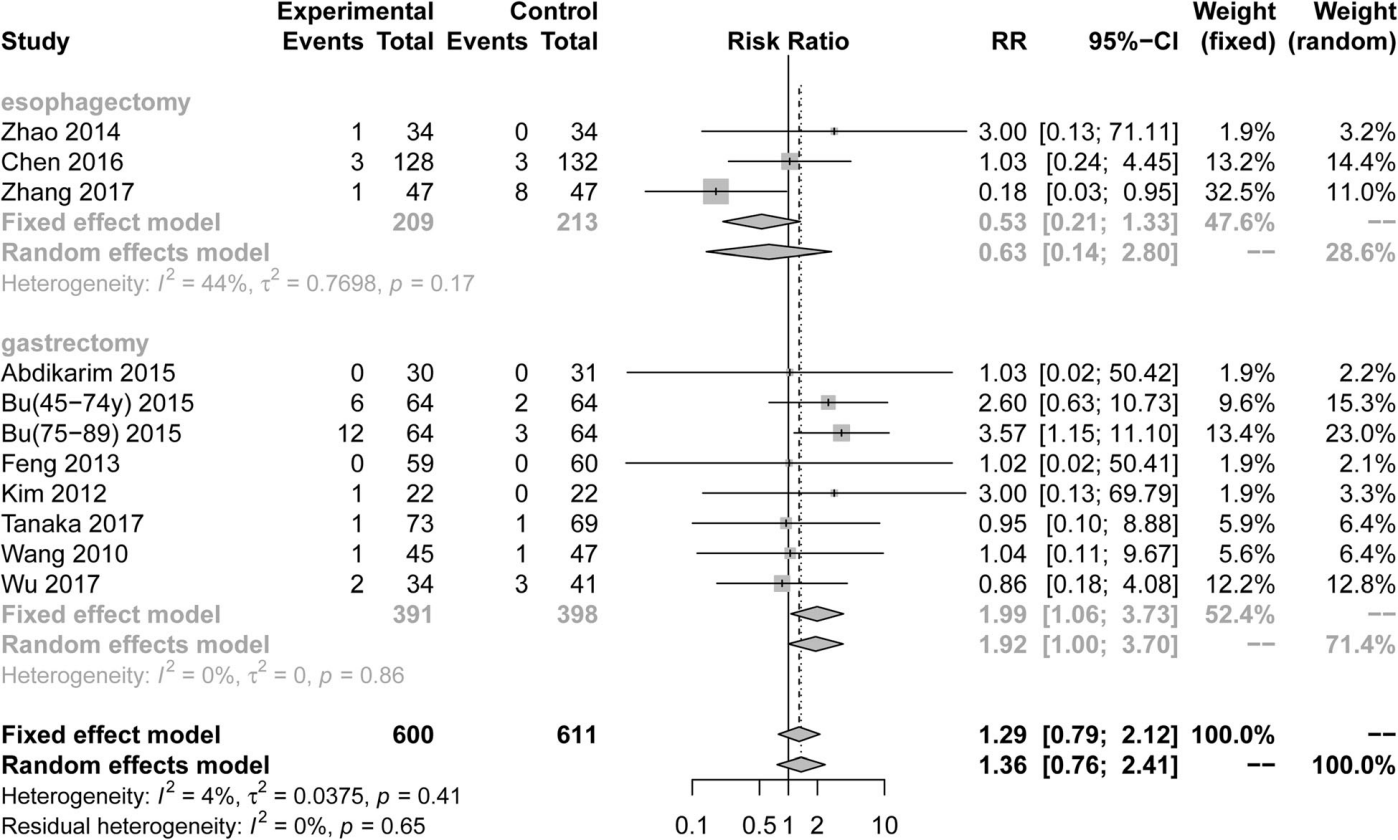
Forest plot evaluating the incidence of readmission between ERAS and conventional care

Eleven RCTs (1211 patients) reported postoperative readmission rates. Pooling the results suggested that ERAS protocols didnot increase postoperative readmission rates compared to conventional care (Fig. 10, RR = 1.29, 95%CI: 0.79 to 2.12). The test of heterogeneity (I^2^ = 4%) indicated that there was little heterogeneity among these trials.

Among RCTs performed in the area of gastric surgery, analysis indicated readmission was significantly increased by ERAS protocols (Fig. 10, RR = 1.99, 95%CI: 1.06 to 3.73, I^2^ = 0%). Among RCTs performed in the area of esophagectomies, readmission rates were not increased by ERAS protocols (Fig. 10, RR = 0.63, 95%CI: 0.14 to 2.80, I^2^ = 44%). However, the results that there were no statistical differences were found in all subgroup analyses of readmission rates based on the surgical procedure and scope of gastrectomy (Table 3).

### Publication bias

There was no evidence of asymmetry in these Doi plots (LFK index < |2|) of the risk of postoperative LI, UTI, SSI and postoperative anastomotic leakage. However, the obvious publication bias was observed in postoperative ileus.

## Discussion

This is a meta-analysis of ERAS protocols in all upper gastrointestinal surgeries. It is also one of the largest studies of upper gastrointestinal RCTs to date. The analysis that we performed indicates that ERAS protocols significantly decreased the incidence of postoperative LI compared to conventional care. ERAS protocols did not increase the incidence of postoperative UTI, SSI, ileus and anastomotic leakage in patients who underwent upper gastrointestinal surgery. Nonetheless, ERAS protocols accelerated patients’ postoperative recovery times.

In all invasive surgeries, the postoperative infection (POI) is a common cause of harm to the patients during the recovery period. A systematic review and meta-analysis [35] indicated that ERAS protocols significantly decreased POI. Most of the ERAS protocol items were included in the studies. Daily smokers have an increased risk of lung infection [36] and postoperative surgical site infection [37]. Preoperative optimization, smoking cessation and refraining from drinking, implementing epidural analgesia [38], fluid management [39], and early mobilization [40] are all steps that can be taken to reduce the incidence of lung infections. ERAS protocols have created a good environment for avoiding postoperative UTI, such as using antimicrobial prophylaxis, maintaining fluid balance [39], and avoiding urinary catheters or removing them early [41]. Non-opioid analgesics can be used to prevent urinary retention. A meta-analysis indicated [42] that the epidural analgesia provided significant improvement in postoperative pain control compared to opioid analgesia. Moreover, using epidurals can decrease insulin resistance. Antimicrobial prophylaxis can be used prior to making a skin incision to prevent SSI [43]. Intake of carbohydrates up to 2 h before anaesthesia does not increase rates of delayed gastric emptying and is recommended prior to surgery [44]. Other practices [6, 34] in ERAS protocols include the use of preoperative carbohydrates, procedures to avoid hypothermia, complete avoidance of the use of nasogastric tubes, and early oral feeding and mobilization.

Traditionally, oral intake was delayed after gastrointestinal surgery to protect anastomoses and prevent postoperative ileus. Early oral feeding in ERAS protocols may increase the risk of vomiting [45]. However, three studies have shown that early oral feeding is not only safe, but it is beneficial to the process of functional recovery [46–48]. A decreased of postoperative gastrointestinal paralysis and ileus was found for epidural anaesthetic compared with epidural opioids in one Cochrane review [49]. Most of the studies we included used early oral feeding, we included more RCTs demonstrating that the use of ERAS does not increase rates of postoperative ileus.

More importantly, this meta-analysis showed that ERAS maybe increased postoperative ileus and readmission rates in patients who underwent upper gastrointestinal surgery, but these failed to reach significance (*P* > 0.10). In the gastrectomy analysis of postoperative ileus rates, we found that the incidence of postoperative ileus in two studies [21, 23] were significantly higher than other studies. The age of the included population in both studies was over 65 years old, it is also the highest in all studies. We exclude these two studies, we found no difference in the incidence of postoperative ileus compared with conventional care. Therefore, according to the available evidence indicates that we should carefully consider whether ERAS should be used in elderly patients undergoing gastric cancer surgery.

We included more studies and found that readmission rates did not increase for all Upper Gastrointestinal Surgeries as a result of ERAS. However, a meta-analysis [10] revealed that ERAS protocols increased the postoperative readmission rates for gastric surgery patients. This is a completely different result from previous meta-analyses [50, 51]. Our subgroup analysis showed that the readmission rates of gastric surgery patients also increased. Excluding the elderly patients in Bu’s study [21] indicated that there was no increase in readmission rates between ERAS and conventional care groups. A RCT [23] showed ERAS combined with laparoscopic surgery can accelerate postoperative recovery time, reduce postoperative stress reaction for elderly GC patients. We tried to explore the benefits of laparoscopy combined with ERAS in the elderly, but the available RCTs too few. RCTs with large studies are needed to more precisely evaluate ERAS in elderly patients. PLOS was shortened 2.68 days and time until the first flatus was shortened by 0.71 days by using ERAS protocols compared to conventional care. These present results are consistent with a previous analysis that was reported by Siotos [9]. Our research also confirms that other than the postoperative length of stay and intestinal function recovery, ERAS protocols does not have a large impact on the scope and surgical procedure of gastrectomy. Compared with laparoscopic surgery, ERAS protocols is more profitable in open surgery. Of course, it is possible that the characteristics of laparoscopic surgery with less trauma and faster recovery make it difficult for ERAS protocols to give full play to its own efficacy. Finally, the accuracy of this result may also be caused by the small sample after being divided by the subgroup ananlysis, which needs to be verified by a larger sample study.

This study provides more evidence to support the published guidelines [6, 34]. A meta-analysis [52] of observational studies for ERAS protocols used in esophagectomies. Six retrospective studies have assessed ERAS protocols for patients who have undergone an esophagectomy. We assessed ERAS for esophagectomies across five RCTs. This meta-analysis indicated that ERAS can shorten PLOS without increasing morbidity and postoperative complications for esophagectomies. Consensus guidelines for enhanced recovery after gastrectomy [6] indicated the quality of current evidence varies substantially and further research need to improve the strength of evidence. The current consensus on the use of ERAS in esophageal surgery [34] does not indicate the benefits of ERAS implementation due to the lack of available research. Our research provides strength of evidence regarding RCTs to support the publication of consensus guidelines on the use of ERAS in gastrectomy and esophagectomy.

Without adoubt, there are several limitations in this study. First, there have been too few RCTs conducted in the area of esophagectomies. Second, there was heterogeneity for PLOS and the time until the first postoperative flatus and defecation. This compelling heterogeneity may be attributable to clinical factors, including the technical skill level of the hospital and surgeon, surgical procedures, and conflicting evaluation of the outcomes. Furthermore, we found that the different types and purposes of surgery, including gastric resection for cancer and sleeve gastrectomy for obesity, were also centralized pooled, which may increase some clinical heterogeneity. In addition, there was not uniform implementation of ERAS recommendations. For example, there were different standards for liquid management documented. Despite attempting to find these types of variations through subgroup analysis, we were unable to detect the source of the heterogeneity. The postoperative ileus rates showed major asymmetry in the Doi plots as evidence of publication bias, which may affect the pooled results. Finally, since many of these studies do not use appropriate randomisation procedures and do not report their methods for from chinese-language studies, there may be potential language bias in this study based on select only English-language studies.

## Conclusions

This study found that ERAS programmes are associated with a significant reduction in postoperative LI, Meanwhile ERAS protocols accelerate patients’ postoperative recovery times included the first postoperative flatus, defecation and PLOS, regardless of the scope and surgical procedure of gastrectomy. We also found that perform ERAS may increase the risk of postoperative ileus or readmission rate. Although, to be truly effective interventions, as the ultimate quality improvement practice, is the key to measuring the success of ERAS, our research provides some reference evidence to advance ERAS into clinical practice.

## Supplementary information

**Additional file 1.** Search strategy.

**Additional file 2.** Reasons for excluding studies by reading full-text.

**Additional file 3.** Assessment of risk of bias.

## Data Availability

Not applicable.

## Abbreviations

CI: Confidence intervals
ERAS: Enhanced recovery after surgery
LI: Lung infection
MD: Mean difference
PLOS: Postoperative length of stay
POI: Postoperative infection
RCT: Randomized controlled trial
REM: Random effects model
SSI: Surgical site infection
UTI: Urinary tract infection
