# Supplementary material for: The application of enhanced recovery after surgery for upper gastrointestinal surgery: Meta-analysis
Source: BMC Surg. 2020 Jan 3;20:3. doi: 10.1186/s12893-019-0669-3 (PMC6942370; doi:10.1186/s12893-019-0669-3)
Supplement: Supplementary file 1 — Additional file 1. Search strategy. [file 12893_2019_669_MOESM1_ESM.docx]

**Additional file 1** Search strategy

**1. Ovid MEDLINE(R) Epub Ahead of Print, In-Process & Other Non-Indexed Citations, Ovid MEDLINE(R) Daily, Ovid MEDLINE and Versions(R),** 1946 to December 12, 2018

1. (Enhanced Recovery After Surgery).mp.
2. (Enhanced Recovery Pathways).mp.
3. (ERAS).mp.
4. (Fast-track surgery).mp.
5. (FTS).mp.
6. (Clinical pathway).mp.
7. (Critical pathway).mp.
8. (Multimodal perioperative care).mp.
9. (Accelerated rehabilitation).mp.
10. 1 or 2 or 3 or 4 or 5 or 6 or 7 or 8 or 9
11. (Digestive System Neoplasms).mp.
12. (Digestive System Surgical Procedures).mp.
13. (Gastrointestinal Neoplasm).mp.
14. 11 or 12 or 13
15. (Esophageal Neoplasms).mp.
16. (Esophageal Cancer).mp.
17. (Esophageal Diseases).mp.
18. (Esophageal Resection).mp.
19. 15 or 16 or 17 or 18
20. (Stomach Neoplasms).mp.
21. (Gastric Cancer).mp.
22. (Stomach Carcinoma).mp.
23. (Gastrectomy).mp.
24. (Pyloromyotomy).mp.
25. (Gastric Resection).mp.
26. (Anastomotic Leak).mp.
27. 20 or 21 or 22 or 23 or 24 or 25 or 26
28. 14 or 19 or 27
29. 10 and 28

**2. OVID Embase Classic+Embase,** 1947 to 2018 December 12

1. (Enhanced Recovery After Surgery).mp.
2. (Enhanced Recovery Pathways).mp.
3. (ERAS).mp.
4. (Fast-track surgery).mp.
5. (FTS).mp.
6. (Clinical pathway).mp.
7. (Critical pathway).mp.
8. (Multimodal perioperative care).mp.
9. (Accelerated rehabilitation).mp.
10. 1 or 2 or 3 or 4 or 5 or 6 or 7 or 8 or 9
11. (Digestive System Neoplasms).mp.
12. (Digestive System Surgical Procedures).mp.
13. (Gastrointestinal Neoplasm).mp.
14. 11 or 12 or 13
15. (Esophageal Neoplasms).mp.
16. (Esophageal Cancer).mp.
17. (Esophageal Diseases).mp.
18. (Esophageal Resection).mp.
19. 15 or 16 or 17 or 18
20. (Stomach Neoplasms).mp.
21. (Gastric Cancer).mp.
22. (Stomach Carcinoma).mp.
23. (Gastrectomy).mp.
24. (Pyloromyotomy).mp.
25. (Gastric Resection).mp.
26. (Anastomotic Leak).mp.
27. 20 or 21 or 22 or 23 or 24 or 25 or 26
28. 14 or 19 or 27
29. 10 and 28

**3. CENTRAL, The Cochrane Library,** 1947 to 2018 December 12

1. Enhanced Recovery After Surgery
2. Enhanced Recovery Pathways
3. ERAS
4. Fast-track surgery
5. FTS
6. Clinical pathway
7. Critical pathway
8. Multimodal perioperative care
9. Accelerated rehabilitation
10. #1 or #2 or #3 or #4 or #5 or #6 or #7 or #8 or #9
11. Digestive System Neoplasms
12. Digestive System Surgical Procedures
13. Gastrointestinal Neoplasm
14. #11 or #12 or #13
15. Esophageal Neoplasms
16. Esophageal Cancer
17. Esophageal Diseases
18. Esophageal Resection
19. #15 or #16 or #17 or #18
20. Stomach Neoplasms
21. Gastric Cancer
22. Stomach Carcinoma
23. Gastrectomy
24. Pyloromyotomy
25. Gastric Resection
26. Anastomotic Leak
27. #20 or #21 or #22 or #23 or #24 or #25 or #26
28. #14 or #19 or #27
29. #10 and #28

**4. ISIWeb of Science,** 1947 to 2018 December 12

#1 TS=((Enhanced Recovery After Surgery) OR (Enhanced Recovery Pathways) OR ERAS OR (fast-track surgery) OR FTS OR (accelerated rehabilitation))

#2 TS=((Digestive System Neoplasms) OR (Digestive System Surgical Procedures) OR (Gastrointestinal Neoplasms))

#3 TS=((Esophageal Neoplasms) OR (Esophageal Cancer) OR (Esophageal Diseases) OR (Esophageal Resection))

#4 TS=((Stomach Neoplasms) OR (Gastric Cancer) OR (Stomach Carcinoma) OR (Gastrectomy) OR (Pyloromyotomy) OR (Gastric Resection) OR (Anastomotic Leak))

#5 #2 OR #3 OR #4

#6 #1 AND #5
