# Supplementary material for: The application of enhanced recovery after surgery for upper gastrointestinal surgery: Meta-analysis
Source: BMC Surg. 2020 Jan 3;20:3. doi: 10.1186/s12893-019-0669-3 (PMC6942370; doi:10.1186/s12893-019-0669-3)
Supplement: Supplementary file 2 — Additional file 2. Reasons for excluding studies by reading full-text. [file 12893_2019_669_MOESM2_ESM.docx]

**Additional file 2** Reasons for excluding studies by reading full-text

| **ID** | **First Author** | **Year** | **Tittle** | **Reasons** |
| --- | --- | --- | --- | --- |
| 1 | XX Liu | 2016 | “Fast‑track” and “Minimally Invasive” Surgery for Gastric Cancer | This is a retrospective study |
| 2 | SH Jeong | 2011 | Is the critical pathway effective for the treatment of gastric cancer? | This is a retrospective study |
| 3 | T Yamada | 2012 | Usefulness of enhanced recovery after surgery protocol as compared with conventional perioperative care in gastric surgery | This is a retrospective study |
| 4 | MR Sahoo | 2014 | Early rehabilitation after surgery program versus conventional care during perioperative period in patients undergoing laparoscopic assisted total gastrectomy | This is a retrospective study |
| 5 | F Fang | 2016 | Effect and clinical significance of fast-track surgery combined with laparoscopic radical gastrectomy on the plasma level of vascular endothelial growth factor in gastric antrum cancer | This is a retrospective study |
| 6 | makuuchi | 2016 | Enhanced recovery after surgery for gastric cancer and an assessment of preoperative carbohydrate loading | This is a case-control study |
| 7 | JB So | 2008 | Reduction of hospital stay and cost after the implementation of a clinical pathway for radical gastrectomy for gastric cancer | Control group is pre-pathway |
| 8 | J zhao | 2018 | Patients Administered Neoadjuvant Chemotherapy Could be Enrolled into an Enhanced Recovery after Surgery Program for Locally Advanced Gastric Cancer | patients who received neoadjuvant chemotherapy |
| 9 | V Munitiz | 2010 | Effectiveness of a written clinical pathway for enhanced recovery after transthoracic (Ivor Lewis) oesophagectomy | This is a retrospective study |
| 10 | C Li | 2012 | An enhanced recovery pathway decreases duration of stay after esophagectomy | This is a retrospective study |
| 11 | S Cao | 2012 | Fast-track rehabilitation program and conventional care after esophagectomy: a retrospective controlled cohort study | This is a retrospective controlled cohort study |
| 12 | L Lee | 2013 | Economic impact of an enhanced recovery pathway for oesophagectomy | This is a retrospective study |
| 13 | RL Blom | 2013 | Initial Experiences of an Enhanced Recovery Protocol in Esophageal Surgery | This is a retrospective study |
| 14 | Huaguang Pan | 2014 | Use of a fast-track surgery protocol on patients undergoing minimally invasive oesophagectomy: preliminary results | This is a retrospective controlled cohort study |
| 15 | JB Shewale | 2015 | Impact of a Fast-Track Esophagectomy Protocol on Esophageal Cancer Patient Outcomes and Hospital Charges | This is a case-control study |
| 16 | Y Akiyama | 2017 | Effectiveness of intervention with a perioperative multidisciplinary support team for radical esophagectomy | This is a retrospective study |
| 17 | S Giacopuzzi | 2017 | Enhanced recovery after surgery protocol in patients undergoing esophagectomy for cancer: a single center experience | This is a historical prospective cohort study |
| 18 | W Raue | 2004 | ‘‘Fast-track’’ multimodal rehabilitation program improves outcome after laparoscopic sigmoidectomy | This is a clinical trial |
| 19 | G Balzano | 2008 | Fast-track recovery programme after pancreaticoduodenectomy reduces delayed gastric emptying | This is a retrospective cohort study |
| 20 | JJ French | 2009 | Fast-track management of patients undergoing proximal pancreatic resection | This is a retrospective study |
| 21 | HM Abu | 2013 | Implementation of enhanced recovery programme after pancreatoduodenectomy: A single-centre UK pilot study | This is a case-control study |
| 22 | HE Kim | 2014 | Impact of critical pathway implementation on hospital stay and costs in patients undergoing pancreaticoduodenectomy | Control group is pre-pathway |
| 23 | M Braga | 2014 | Enhanced Recovery After Surgery Pathway in Patients Undergoing Pancreaticoduodenectomy | This is a historical prospective cohort study |
| 24 | MM Coolsen | 2014 | Improving Outcome after Pancreaticoduodenectomy: Experiences with Implementing an Enhanced Recovery After Surgery (ERAS) Program | This is a retrospective study |
| 25 | S Kobayashi | 2014 | Perioperative Care with Fast-Track Management in Patients Undergoing Pancreaticoduodenectomy | This is a case-control study |
| 26 | Sastha Ahanatha Pillai | 2014 | Feasibility of implementing fast-track surgery in pancreaticoduodenectomy with pancreaticogastrostomy for reconstruction -A prospective cohort study with historical control | This is a historical prospective cohort study |
| 27 | C Williamsson | 2015 | Impact of a fast-track surgery programme for pancreaticoduodenectomy | This is a case-control study |
| 28 | GR Joliat | 2015 | Cost–benefit analysis of an enhanced recovery protocol for pancreaticoduodenectomy | This is a prospective cohort study |
| 29 | J Richardson | 2015 | Implementation of enhanced recovery programme for laparoscopic distal pancreatectomy: Feasibility, safety and cost analysis | This is a case-control study |
| 30 | Z Shao | 2015 | The role of fast-track surgery in pancreaticoduodenectomy: A retrospective cohort study of 635 consecutive resections | This is a retrospective cohort study |
| 31 | E Zouros | 2016 | Improvement of gastric emptying by enhanced recovery after pancreaticoduodenectomy | This is a case-control study |
| 32 | KA Morgan | 2016 | Enhanced Recovery After Surgery Protocols Are Valuable in Pancreas Surgery Patients | This is a case-control study |
| 33 | OJ Shah | 2016 | Impact of centralization of pancreaticoduodenectomy coupled with fast track recovery protocol: a comparative study from India | This is a case-control study |
| 34 | S Partelli | 2016 | Evaluation of an enhanced recovery protocol after pancreaticoduodenectomy in elderly patients | This is a retrospective study |
| 35 | X Bai | 2016 | The implementation of an enhanced recovery after surgery (ERAS) program following pancreatic surgery in an academic medical center of China | This is a historical prospective cohort study |
| 36 | DJ Kagedan | 2017 | The economics of recovery after pancreatic surgery: detailed cost minimization analysis of an enhanced recovery program | This is a retrospective cohort study |
| 37 | J Dai | 2017 | Reducing postoperative complications and improving clinical outcome: Enhanced recovery after surgery in pancreaticoduodenectomy -A retrospective cohort study | This is a retrospective cohort study |
| 38 | M van der Kolk | 2017 | Implementation and Evaluation of a Clinical Pathway for Pancreaticoduodenectomy Procedures: A Prospective Cohort Study | This is a Prospective Cohort Study |
| 39 | N Pecorelli | 2017 | Enhanced recovery pathway in patients undergoing distal pancreatectomy: a case-matched study | This is a retrospective case-control study |
| 40 | M Gonenc | 2014 | Enhanced postoperative recovery pathways in emergency surgery: a randomised controlled clinical trial | This is a emergency surgery |
| 41 | GH Mannaerts | 2016 | Results of Implementing an Enhanced Recovery After Bariatric Surgery (ERABS) Protocol | This is a historical prospective cohort study |
| 42 | V Simonelli | 2016 | Fast-Track in Bariatric and Metabolic Surgery: Feasibility and Cost Analysis Through a Matched-Cohort Study in a Single Centre | This is a matched-cohort study |
| 43 | J Ruiz-Tovar | 2018 | Implementation of the Spanish ERAS program in bariatric surgery | This is a historical prospective cohort study |
| 44 | Ma J | 2014 | Application and effect of accelerated rehabilitation surgical care in perioperative nursing in digestive system tumors | Full-text language chinese |
| 45 | You, Z. B. | 2012 | Application of fast-track surgery in the management of patients with esophageal cancer | Full-text language chinese |
| 46 | JY Wang | 2015 | Application of fast-track surgery in the management of nutritional risk on patients with esophageal carcinoma after esophagectomy perioperatively | Full-text language chinese |
| 47 | Ling, F. Y. | 2017 | Value of enhanced recovery after surgery in perioperative period of minimally invasive esophagectomy for esophageal carcinom | Full-text language chinese |
| 48 | ZW Jiang | 2007 | The safety and efficiency of fast track surgery in gastric cancer patients undergoing D2 gastrectomy | Full-text language chinese |
| 49 | DS Wang | 2009 | Observation of fast track surgery in patients with gastric cancer | Full-text language is chinese |
| 50 | Y Tang | 2010 | Clinical appficafion of perioperative fast-track and nutrition support program in elderly patients with gastric cancer | Full-text language is chinese |
| 51 | ZG, He | 2010 | Benefits of perioperative fast-track surgery program on clinical outcome in patients with gastric cancer | Full-text language is chinese |
| 52 | Xie, Z. Y. | 2012 | Effect of fast track surgery on clinical parameters and postoperative complications in patients with gastric cance | Full-text language is chinese |
| 53 | G Wang | 2014 | Promotion of postoperative recovery with fast track surgery for gastric cancer patients undergoing gastrectomy: a prospective randomized controlled study | Full-text language is chinese |
| 54 | Yang Y | 2015 | Application of enhanced recovery program after surgery in patients undergoing elective radical resection for gastric cancer | Full-text language is chinese |
| 55 | Li, Y. P | 2016 | Application of enhanced recovery after surgery for patients with laparoscopic radical gastrectom | Full-text language is chinese |
| 56 | X Xu | 2017 | Application of enhanced recovery after surgery in laparoscopy-assisted distal gastrectomy | Full-text language is chinese |
| 57 | Zhang, X. F | 2018 | Effect of fast track surgery concept based nursing intervention on rehabilitation and nursing satisfaction in patients with advanced gastric cancer after laparoscopic assisted D2 radical operation | Full-text language is chinese |
| 58 | Ding, Y. | 2017 | Application of enhanced recovery program in laparoscopic distal pancreatectomy | Full-text language is chinese |
| 59 | S.J. Ford | 2014 | The implementation and effectiveness of an enhanced recovery programme after oesophago-gastrectomy: A prospective cohort study | This is a prospective cohort study |
| 60 | Koyama | 2000 | Improvement of the efficiency of the treatment of gastric cancer by the standardization of the treatment plan | Full-text language is Japanese |
| 61 | T Kiyama | 2003 | Clinical significance of a standardized clinical pathway in gastrectomy patients | Full-text language is Japanese |
| 62 | T Kiyama | 2003 | An economic evaluation on the clinical pathway for gastrectomy | Full-text language is Japanese |
| 63 | Kovalenko, Z. A | 2017 | Accelerated postoperative rehabilitation in patients undergoing pancreatoduodenectomy | Full-text language is Russian |
| 64 | Morales Soriano, R. | 2015 | Outcomes of an enhanced recovery after surgery programme for pancreaticoduodenectomy | Full-text language is Russian |
| 65 | R Bhandari | 2015 | Implementation and Effectiveness of Early Chest Tube Removal during an Enhanced Recovery Programme after Oesophago-gastrectomy | No available outcomes data |
| 66 | Pimenta GP | 2015 | Sleeve Gastrectomy With or Without a Multimodal Perioperative Care. A Randomized Pilot Study | No available outcomes data |
| 67 | Zang YF | 2018 | Application value of enhanced recovery after surgery for total laparoscopic uncut Roux-en-Y gastrojejunostomy after distal gastrectomy | This is a retrospective cohort study |
| 68 | Lemanu DP | 2013 | Randomized clinical trial of enhanced recovery versus standard care after laparoscopic sleeve gastrectomy | This is a sleeve gastrectomy for obesity. |
