# Supplementary material for: The application of enhanced recovery after surgery for upper gastrointestinal surgery: Meta-analysis
Source: BMC Surg. 2020 Jan 3;20:3. doi: 10.1186/s12893-019-0669-3 (PMC6942370; doi:10.1186/s12893-019-0669-3)
Supplement: Supplementary file 3 — Additional file 3. Assessment of risk of bias. [file 12893_2019_669_MOESM3_ESM.docx]

**Additional file 3** Assessment of risk of bias

|  | **Random sequence generation (Selection bias)** | **Allocation concealment (Selection bias)** | **Blinding of participants and personnel (Performance bias)** | **Blinding of outcome assessment (Detection bias)** | **Incomplete outcome data (Attrition bias)** | **Selective reporting (Reporting bias)** | **Other bias** |
| --- | --- | --- | --- | --- | --- | --- | --- |
| **Wang 2010 [18]** | ? | ? | - | ? | + | + | + |
| **Chen (LS) ‎2012 [19]** | ? | + | - | + | + | + | + |
| **Chen (OS) ‎2012 [19]** | ? | + | - | + | + | + | + |
| **Feng 2013 [20]** | + | + | - | ? | + | + | + |
| **Bu (45-74y) 2015 [21]** | + | ? | - | - | + | + | + |
| **Bu (75-89y) 2015 [21]** | + | ? | - | - | + | + | + |
| **Abdikarim 2015 [22]** | ? | + | - | + | ? | + | + |
| **Liu (LS) 2016 [23]** | + | ? | - | ? | ? | + | - |
| **Liu (OS) 2016 [23]** | + | ? | - | ? | ? | + | - |
| **Fujikuni 2016 [24]** | ? | ? | - | ? | ? | + | - |
| **Tanaka 2017 [25]** | + | - | - | + | + | + | + |
| **Xia 2017 [26]** | + | ? | - | ? | + | + | + |
| **Wu 2017 [27]** | ? | ? | - | ? | ? | + | + |
| **Kim 2012 [28]** | + | + | - | ? | + | + | + |
| **Zhao 2014 [29]** | + | + | - | ? | + | + | + |
| **Chen 2016 [30]** | + | ? | - | ? | + | + | + |
| **Li 2017 [31]** | ? | ? | - | ? | ? | + | + |
| **Zhang 2017 [32]** | ? | ? | - | ? | ? | + | + |
| **Zhang 2018 [33]** | ? | ? | - | ? | ? | + | + |

**Note**: LS: Laparoscopic surgery; OS: open surgery; 45-74y: Patients aged 45-74 years; 75-89y: Patients aged 75-89 years. Assessment of bias in randomized trials.

+ (green) denotes low risk of bias, – (red) denotes high risk of bias. ? (yellow) denotes uncertain risk of bias.
